# Behind the clock: elucidating factors contributing to longer clinic appointment duration and patient wait time

**DOI:** 10.1186/s12913-021-06079-y

**Published:** 2021-01-26

**Authors:** Daniel Jonathan Kagedan, Stephen B. Edge, Kazuaki Takabe

**Affiliations:** 1 Department of Surgical Oncology, Roswell Park Comprehensive Cancer Center, Buffalo, NY USA; 2 Jacobs School of Medicine and Biomedical Sciences, University of Buffalo, Buffalo, NY USA; 3 Roswell Park Comprehensive Cancer Center, Clinical Sciences Center, Room P-615 Elm and Carlton Streets, 14263 Buffalo, NY USA

**Keywords:** Breast surgery, Breast cancer, Surgical training, Ambulatory care, Time factors

## Abstract

**Background:**

Longer wait time in ambulatory clinics can disrupt schedules and decrease satisfaction. We investigated factors associated with patient wait time (WT, check-in to examination room placement), approximate clinician time (ACT, completion of nurse assessment to check-out), and total appointment length (TAL, check-in to check-out).

**Methods:**

A single-institution retrospective study was conducted of breast surgery clinic patients, 2017–2019, using actual encounter times. A before/after analysis compared a five-day 8 hour/day (from a four-day 10 hour/day) advanced practice provider (APP) work-week. Non-parametric tests were used, and medians with interquartile ranges (IQRs) reported.

**Results:**

15,265 encounters were identified. Overall WT was 15.0 minutes (IQR:6.0–32.0), ACT 49.0 minutes (IQR:31.0–79.0) and TAL 84.0 minutes (IQR:57.0-124.0). Trainees were associated with 30.0 minutes longer ACT (*p* < 0.0001); this increased time was greatest for follow-up appointments, least for new patients. Patients arriving > 5 minutes late (versus on-time) experienced shorter WT (11.0 vs. 15.0 minutes, *p* < 0.0001) and ACT (43.0 vs. 53.0 minutes, *p *< 0.0001). Busier days (higher encounter volume:APP ratios) demonstrated increased encounter times. After transitioning to a five-day APP work-week, ACT decreased.

**Conclusions:**

High-volume clinics and trainee involvement prolong ambulatory encounters. Increasing APP assistance, altering work schedules, and assigning follow-up appointments to non-trainees may decrease encounter time.

## Background

Ambulatory clinic encounters are a key component of surgical and oncology practice. Providers generally strive to be punctual, adhering to their schedule and seeing patients promptly at their designated appointment time. However, many clinics run behind schedule, disrupting schedules for patients, families, and healthcare providers [1]. Longer wait time has been linked to decreased patient satisfaction and poorer patient perception of care provided [2–7]. Numerous factors have been postulated to contribute to increased clinic appointment time, and in turn to longer clinic wait time. These include trainee involvement in appointments [8], patients arriving late for their scheduled encounters [9], and busier clinics with higher daily volumes of appointments scheduled and fewer experienced clinical assistants (i.e. advanced practice providers or APPs, such as nurse practitioners or physician assistants) [10]. We sought to investigate the influence of specific predetermined factors on various clinic encounter times including the total appointment duration, the wait time, and the time spent with clinician(s) in an outpatient breast surgery clinic at a high-volume cancer center. We hypothesized that trainee involvement, late patient arrivals, and increased ratios of daily clinic volume to clinical assistant would be associated with increased encounter time duration. Furthermore, during the study period the APPs transitioned from working four days weekly (10 hours daily) to five days weekly (8 hours daily) with resultant greater availability of APPs during the busiest times. This initiative was spearheaded by the Director of Practice Administration at Roswell Park Comprehensive Cancer Center with the goal of optimizing coverage and improving scheduling. We hypothesized that the five-day APP schedule would be associated with decreased encounter duration compared to the former.

## Methods

### Study cohort and database description

This study was conducted at a single institution using a retrospective cohort design utilizing a database of ambulatory breast surgery clinic encounters. Ambulatory (outpatient) clinic encounters (used interchangeably with appointments) at a breast surgical oncology center occurring from January 1, 2017 to May 21, 2019 were identified and tracking data from these encounters included in this study. The clinic utilizes a real-time patient tracking system to record every clinic encounter. Data captured include the unique patient medical record number (MRN) associated with it, and the specific clock times of various components of each encounter, rounded to the nearest minute. The encounter times analyzed in this study are recorded by clinic registration staff (check in time, check out time), and clinical nursing staff (patient placed in room time, end of nursing assessment time). Times are used interchangeable with durations and are reported herein in minutes. Also recorded for each encounter using the patient tracking system are the involvement of trainees, as well as the name of the APP and/or attending MD who saw the patient.

The study took place at a single institution, Roswell Park Comprehensive Cancer Center, and used data from the breast surgery clinic, which employs 5 attending surgeons who see outpatients in this setting. The clinic operates from 8 AM to 5 PM, Monday to Friday. Each attending surgeon holds clinic 1–2 days per week for scheduled visits, with infrequent urgent appointments throughout the week as needed. There is generally only one attending physician and 1–4 APPs in the clinic at any given time. APP encounters are either under the supervision of an attending physician (APP sees patient first, then attending MD sees patient), or independently (APP alone sees patient) for certain pre-determined designated appointment types including surveillance, certain high-risk follow-ups, and unscheduled post-operative checks. Additionally, trainees including post-graduate year (PGY)-2 or PGY-3 general surgery residents, complex general surgical oncology fellows, and breast fellows attend clinic and see patients, always under the supervision of the attending surgeon (trainee sees patient first, then attending MD sees patient).

Clinic encounters were classified into 6 categories based on referral details (new patients) and/or patient history (established patients) as follows: new malignant (new diagnosis of cancer); new benign (new referrals for benign disease); new high risk (new referrals for high risk screening/treatment); pre-operative (established patients planning for operative intervention); post-operative (established patients returning for their first appointment following operative intervention); and follow-up (established patients undergoing surveillance, high risk screening, treatment monitoring). Patients seen for urgent assessments (i.e. sick visits) were grouped into an “other” category. Encounters seen solely by APPs were designated accordingly. The classification of the type of clinic encounter is compiled beforehand in a daily appointment list. The appointment time is also recorded on the appointment list. Institutional Research Board approval was obtained from the institutional ethics board at Roswell Park Comprehensive Cancer Center (reference number: 00001124).

### Clinic encounter times and outcome variables

Patient flow through a clinical encounter proceeds as follows. The patient arrives and registers at the clinic check-in (check-in time). Patients not previously seen at Roswell Park are asked to present to the institutional registration (separate from the clinic check-in) one hour before the scheduled appointment time. Follow-up patients may also have appointments for breast imaging prior to the breast clinic visit. The time allotted for institution registration and for breast imaging were not included in this study. Patients are then called from the waiting room into the examination room (patient placed in room time) where a clinic registered nurse (RN) assesses them, including recording their vital signs and asking questions related to medication use, distress level, and other social and medical issues. The interval between check-in time and patient placed in room time is designated as “waiting time” (WT). Depending on the type of visit and patient’s situation, the subsequent nursing assessment takes from 5 to 30 minutes. After the nursing assessment is complete, the patient’s chart is placed in the clinician work room, and the patient’s assessment by the clinician (attending MD, APP, or trainee) begins (beginning of clinical encounter time). Of note, the tracking board records the time at which the patient is ready for the clinician. Therefore for this study the conclusion of the nursing assessment was used instead to approximate the beginning of the clinician assessment, and the time of departure from the clinic as the end of the clinician assessment. The patient is seen by either the attending MD alone, an APP alone, or a combination of trainee or APP followed by attending MD. The choice depends on the work flow of the day, provider availability, and patient factors. Following the clinical assessment, the patient checks out of clinic (check-out time) and departs. If a patient is sent for additional testing (i.e. bloodwork, imaging) during their encounter, this is noted and the time from the patient’s departure from clinic until their return is subtracted from their corresponding encounter times. Because clinicians do not reliably record in the system the time of the start or end of their clinical encounter, the interval between the end of the nursing assessment and the check-out time is designated as “approximate clinician time” (ACT). Of note, this includes the time that a patient is ready for the clinician but waiting for a clinician to be available, the time spent by the clinician reviewing the chart prior to entering the patient room, as well as the time for scheduling follow-up appointments and other ancillary services performed in the clinic visit. The interval between check-in time and check-out time is designated as “total appointment length” (TAL). The three outcome variables (WT, ACT, TAL) were analyzed as continuous variables. Negative values, resulting from missing or incorrectly recorded timestamps, were excluded from analysis. Additionally, ACT and TAL with zero values were excluded.

### Early and late patient arrivals

To analyze the effects of early and late patient arrival on clinic encounter times, the difference between the scheduled appointment time and the patient check-in time (actual arrival time) was calculated for each encounter. Negative and missing values were excluded. The remaining values were transformed into a 3-level categorical variable, in which “early” denoted patient arrival more than 15 minutes prior to the scheduled appointment time, “on time” denoted arrival between 15 minutes prior to 5 minutes after the scheduled appointment time, and “late” denoted arrival more than 5 minutes after the scheduled appointment time. Of note, the pre-appointment information provided to patients recommends arriving 15 minutes prior to their scheduled appointment time.

### Clinic volume and number of APPs working

The number of APPs seeing patients in clinic on a given day was calculated as the sum of the unique names of APPs recorded as seeing at least one clinic patient on the day in question. The number of clinic encounters on a given day was determined as the sum of encounters for each calendar date, with no shows excluded. To analyze the effects of both APP number and daily encounter volume on encounter times, the ratio of daily clinic encounters to number of APPs working was calculated for each date. This was then transformed into an ordinal variable with cut points defined at each whole number (integer) ratio value. Encounters occurring on days without APPs working in clinic were excluded from the corresponding analyses.

### APP schedule transition

On January 1, 2019, the breast surgery clinic APPs transitioned from working four days per week for 10 hours daily to working five days per week for 8 hours daily. To analyze the effect of this change on study outcomes, the encounter times were compared before and after the date of implementation over a similar time period (Jan. 1-May 22, 2018 vs. Jan. 1-May 22, 2019) to account for possible confounding by different times of year. The number of daily encounters was also compared before and after implementation, to assess for possible confounding.

### Statistical analyses

Non-parametric tests (Mann-Whitney U, Kruskal-Wallis) were used to compare the effects of trainee/APP involvement, daily clinic encounter volume, and the influence of late patient arrivals on WT, ACT, and TAL. Results were presented as median values with inter-quartile ranges (IQRs), and graphically represented in the Figures by an error bar demonstrating the 75th percentile value. The correlation of clinic encounter volume:APP number versus encounter times was determined by calculating the coefficient of determination, reported as R^2^. Two-sided hypothesis testing was performed for all statistical procedures, and an alpha of 0.05 was used to establish statistical significance. All analyses were performed using SPSS version 25. The datasets used and/or analyzed during the current study are available from the corresponding author upon reasonable request.

## Results

### Cohort description

**Fig. 1:**
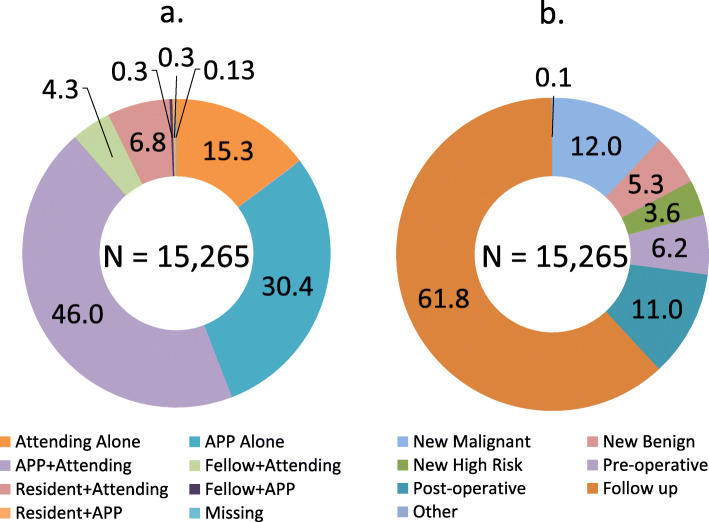
Percentage of clinic appointments (15,265 total) subdivided by: (**a**) clinician(s) involved; and (**b**) appointment type. Legend: APP = advanced practice provider

Between January 1, 2017 and May 23, 2019, there were 15,265 encounters in the breast surgical clinic. Clinician types and appointment types are presented in Fig. 1. The majority of encounters involved APPs (76.4 %); 46.0 % were seen in combination with the attending MD, and 30.4 % were seen by APPs alone. The most common encounter type was follow-up (61.8 %) appointments.

### Clinic encounter times

After excluding patients with missing values (*N* = 509) and negative values (*N* = 21), the median WT was 15.0 minutes (IQR: 6.0–32.0 minutes). WT values of zero minutes were recorded for 149 patients (1.0 %). WT was 2.0 minutes longer for afternoon compared to morning appointments (16.0 vs. 14.0 minutes, *p* < 0.0001), and 4.0–6.0 minutes longer for appointments in spring, summer, and autumn compared to winter (16.0, 18.0, and 16.0 vs. 12.0 minutes, respectively; *p* < 0.0001).

Median ACT was 49.0 minutes (IQR: 31.0–79.0 minutes), after excluding patients with negative values (*N* = 768) and zero values (*N* = 353). The median TAL was 84.0 minutes (IQR: 57.0-124.0 minutes), after excluding negative values (*N* = 806) and zero values (*N* = 297).

### Impact of trainee involvement on clinic times

**Fig. 2:**
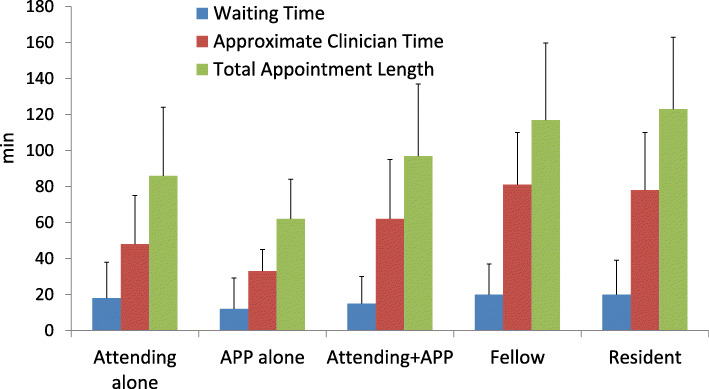
Median encounter times (waiting time, approximate clinician time, total appointment length), stratified by clinician type(s). Legend: Error bars denote 75th percentile values. APP = advanced practice provider

TAL and ACT was shorter for patients seen by a single clinician (attending MD or APP alone), and shortest for APP alone appointments (Fig. 2). Among appointments involving the attending MD, TAL increased significantly when fellows (117.0 minutes, IQR: 87.0-159.75) or residents (123.0 minutes, IQR: 89.0-163.0) were involved, compared to attending MD alone (86.0 minutes, IQR: 60.0-124.0) or attending MD with APP (97.0 minutes, IQR: 67.0-137.0) encounters (*p* < 0.0001) (Fig. 2).

**Fig. 3:**
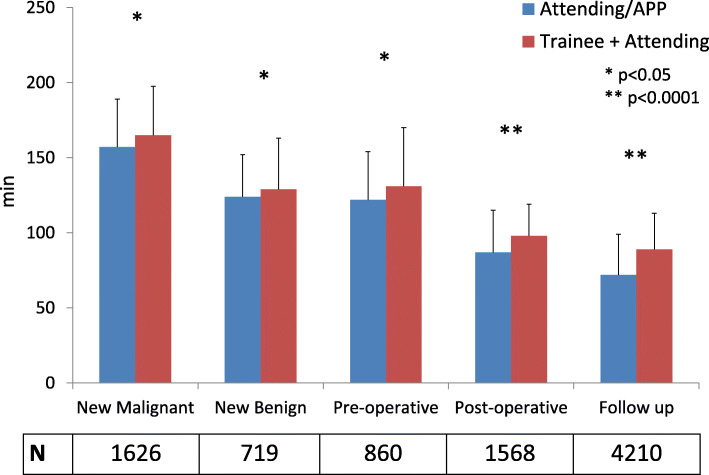
Median total appointment length in minutes for attendings/APPs versus trainees, stratified by appointment type. Legend: Error bars denote 75th percentile values; values below appointment type denote number of encounters included for corresponding appointment type. APP = advanced practice provider. * = *p* < 0.05; ** =* p* < 0.0001

When stratified by encounter type (excluding designated APP encounters), the magnitude of increased TAL associated with trainees was greatest for follow-up (89.0 vs. 72.0 minutes) appointments (*p* < 0.0001), with smaller differences for new patient assessments (Fig. 3). The increased TAL observed with trainee involvement corresponded to increased approximate clinician time for patients seen with trainees compared to attending MDs/APPs alone (76.5 vs. 46.0 minutes, *p* < 0.0001).

### Impact of late patient arrivals on clinic times

**Fig. 4:**
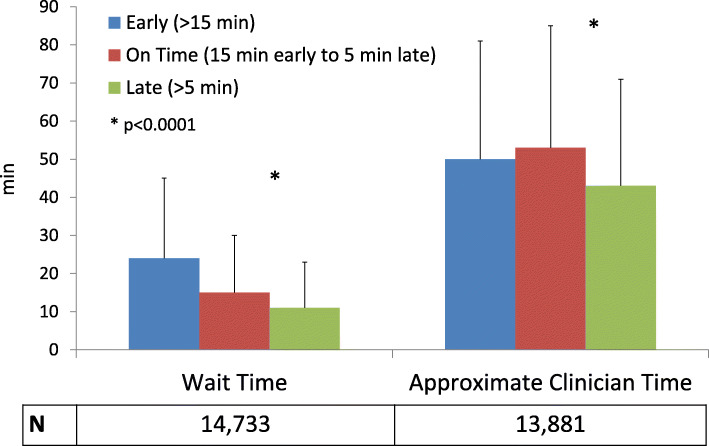
Median waiting time and approximate clinician time, stratified by arrival time type (early, on-time, late). Legend: Error bars denote 75th percentile values; values below encounter time denote number of encounters included for corresponding encounter time. * = *p* < 0.0001 comparing on-time vs. late arrivals

After applying exclusions, patients arrived more than 15 minutes early for 27.1 % of encounters, more than 5 minutes late for 34.3 %, and on time for 38.6 % (*N* = 14,733). Patients were most frequently early for initial assessment of benign disease (35.9 %) and most frequently late for follow-up appointments (36.4 %). WT was shorter for encounters with late arrivals (11.0 minutes, IQR: 5.0–23.0) compared to on-time (15.0 minutes, IQR: 6.0–30.0) and early arrivals (24.0 minutes, IQR: 9.0–45.0; *p* < 0.0001) (Fig. 4). ACT was also shorter for encounters with late patient arrivals (43.0 minutes, IQR: 29.0–71.0) versus on-time and early arrivals (53.0 [IQR: 33.0–85.0] and 50.0 [IQR: 32.0–81.0] minutes, respectively; *p* < 0.0001) (Fig. 4).

### Association of daily clinic volume and APP staffing on clinic times

**Fig. 5:**
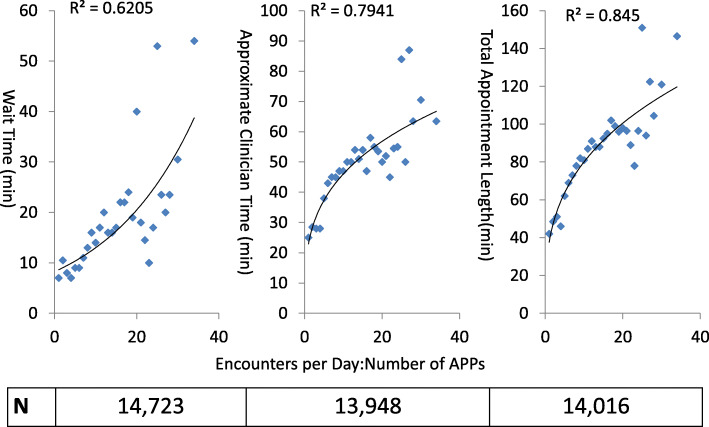
Median encounter times versus ratio of daily clinic encounters to number of APPs in clinic. Legend: Values below charts denote number of encounters included for corresponding analysis. APP = advanced practice provider

Most encounters occurred on days when two APPs were seeing patients in clinic. Approximately 15 % of encounters occurred on days with one or four APPs working. On days with APPs, WT was similar regardless of the number of APPs seeing patients (range: 15.0–19.0 minutes); conversely, ACT was shorter when four APPs were working compared to one to three (44.0 vs. 49.0–50.0 minutes, respectively; *p *= 0.019). The median number of encounters per day was 28.0 (IQR: 22.0–33.0), and the median ratio of encounters per day divided by number of APPs seeing patients on that day was 11.0 (IQR: 9.0–14.0). Days with higher ratios of encounters per APP were associated with longer WT (*R*^2^ = 0.62, *p* < 0.0001), longer ACT (*R*^2^ = 0.79, *p* < 0.0001), and longer TAL (*R*^2^ = 0.84, *p* < 0.0001) (Fig. 5).

### Impact of transition from four- to five-day APP work week on clinic times

**Fig. 6:**
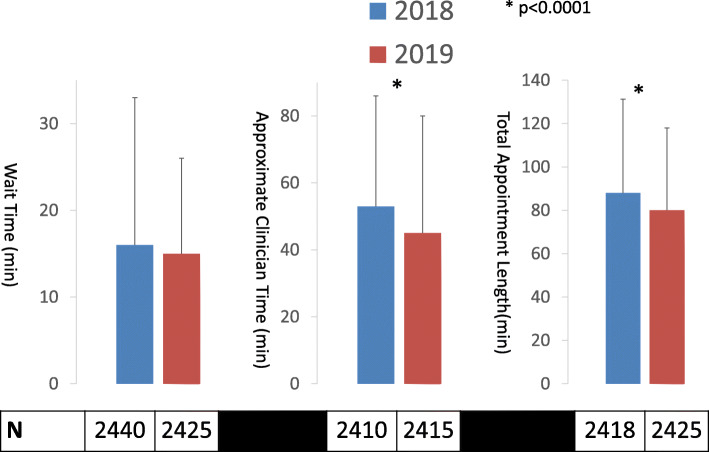
Median encounter times comparing 2018 (4-day APP work week) vs. 2019 (5-day APP work week). Legend: Error bars denote 75th percentile values; values below encounter time denote number of encounters included for corresponding encounter time and year. APP = advanced practice provider. * = *p* < 0.0001

The APPs transitioned from a four-day to five-day work week effective January 1, 2019. Between Jan. 1 – May 22, 2018, there were 2,529 clinic encounters (pre-implementation), compared to 2,512 encounters during the corresponding time period in 2019 (post-implementation). The number of days with four APPs working increased from 9.1 % in 2018 to 39.3 % in 2019, and the number of days with two APPs working decreased from 39.9 % in 2018 to 13.2 % in 2019. The median number of clinic encounters per day was similar before and after intervention implementation (28.0 vs. 28.0; *p* = 0.966). TAL decreased from 88.0 minutes (IQR: 59.0-131.25) to 80.0 minutes (IQR: 57.0-118.0; *p* < 0.0001) following implementation, reflecting a concomitant decrease in ACT from 53.0 minutes (IQR: 33.0–86.0) to 45.0 minutes (IQR: 28.0–80.0; *p* < 0.0001) (Fig. 6). WT remained similar before and after this intervention (16.0 vs. 15.0 minutes; *p* = 0.944) (Fig. 6).

## Discussion

This study reports clinical encounter times for ambulatory breast surgery appointments at an academic cancer center. It identifies an association of trainee involvement with increased appointment duration, most pronounced for established patients. Busier clinics (increased daily encounter volumes) and busy clinics with fewer specialist clinicians (increased daily encounter volume: APPs working that day ratios) were associated with prolonged WT and ACT. Transitioning APPs’ schedule to a five-day work week was associated with decreased ACT (time from the completion of the nursing assessment to clinic check out), but did not alter WT.

The increased ACT observed for encounters involving trainees has previously been reported in the literature [8, 11] although the impact on overall clinic workflow varies [12]. The current study identified this increase to be greatest among encounters for established patients (post-operative, follow-up appointments). This may reflect clinician familiarity with the individual patient, or superior knowledge of clinic processes and diseases enabling more efficient assessment and formulation of management plans compared to trainees. The lack of impact of trainees on new patient times may reflect the longer times spent counseling these patients irrespective of the inclusion of trainees. These findings support assigning new patients preferentially to trainees not only for educational reasons but also to improve clinic efficiency [13], and suggests a potential triage role in encounter type assignment for attending physicians [14].

The association of late patient arrival time with decreased WT and ACT has been previously reported in the literature [9]. The reasons late arrival impacts WT are not understood, and has been suggested to relate to provider flexibility in seeing patients out of their scheduled order [15]. The shorter ACT may relate to the type of encounter at which patients are most often late (follow-up), which are often less complex than other encounter types. Additionally, it is possible that patients who arrive late, especially those being seen for routine follow up appointments, have communication styles more conducive to shorter interactions, although this is purely speculative. An important limitation in the analysis of arrival times relates to same-day imaging occurring prior to scheduled follow-up appointments. Patients are booked for imaging (usually mammography) 90 minutes prior to their scheduled breast surgery clinic follow-up appointment; accordingly, the late arrival in breast surgery clinic may result from delays in obtaining imaging, as opposed to being patient-driven. Further investigation into the influence of pre-appointment imaging on patient clinic arrival time is needed to determine whether follow-up imaging should be scheduled more than 90 minutes prior to the patient’s clinic appointment time.

The association of longer ACT with increased clinic encounter to APP ratio suggests that efficiency is compromised in busier clinics [16]. This finding may also reflect increased time taken in placating patients regarding the longer waiting time, or longer time required at the conclusion of appointments to complete administrative tasks and check out patients when clinics are busier [17]. Notably, the relationship between encounter times and clinic encounter to APP ratio does not demonstrate a clear threshold. This has important implications for clinic scheduling, suggesting that any increase in encounter volume (or decrease in APP number) will prolong encounter times. The variability in encounter times for the busiest clinics may reflect the smaller number of busiest clinic days analyzed.

After APPs transitioned from working four days per week (10 hours per day) to five days per week (8 hours per day), ACT decreased and WT remained unchanged. One possible interpretation of these findings is that APP efficiency increases to ensure that patients are seen and attendant tasks (placing orders, writing notes) completed in less time when the total number of work hours in a day are shortened from ten hours to eight. The unchanged WT implies that the availability of examination rooms in which patients are placed at the end of their WT is not a limiting factor on encounter times and workflow in this clinic. It is not known whether this decreased ACT compromises patient satisfaction, accuracy or completeness of documentation, or patient-clinician communication, due to the lack of data on these outcomes in this study; however these are all potential consequences of the APP schedule transition, and warrant further investigation [18, 19]. Alternatively, the presence of more APPs in the clinic each day may decrease ACT by decreasing the time from completion of the nursing assessment to the clinician seeing the patient [20]. The practice change initiative described in this study could be applied to other clinical settings with a five day work week, such as outpatient ambulatory clinics in other specialties, elective operating room staff and schedulers, and certain allied healthcare professionals such as cancer care navigators and discharge planners. In the authors’ opinion, an optimal change management strategy should begin with soliciting input and suggestions from individuals most impacted by a proposed initiative prior to its implementation. The initiative should then be modified based on the feedback received. Once implemented, iterative cycles of audit and feedback or plan-do-study-act (PDSA) cycles should be regularly scheduled, and steps taken to mitigate any dissatisfaction or unintended negative consequences. The initiative should be evaluated both in terms of quantitative outcome metrics such as waiting time, as well as the results of anonymous surveys of staff and patient satisfaction.

The findings of this study suggest that ambulatory clinic encounter times can potentially be optimized by assigning new patients preferentially to trainees as opposed to follow-up encounters, by increasing the number of APPs especially for higher volume clinics and potentially scheduling fewer visits when fewer APPs are available, and by optimizing APP availability through implementation of a five-day work week. However, any new initiative should be repeatedly evaluated to determine its impact on clinic encounter times, and patient and provider satisfaction, as well as unanticipated negative consequences.

Strengths of the current study include the large number of patient encounters included, the ability to differentiate encounter types for stratification, and the clear definition of arrival time and the end of nursing assessment time. Limitations include the retrospective nature of the study design, and the reliance upon the manually-entered time stamp tracking system data. However, a previous publication comparing time stamp patient tracking systems versus actual patient flow through an ambulatory clinic reported a high level of concordance (within 3 minutes for > 80 % of appointments) [21]. Another limitation relates to the definition used of ACT, from the conclusion of the nursing assessment to the patient’s departure from clinic. This was because more granular information on time spent with the clinician was not reliably recorded. The study definition of ACT may be artificially lengthened at the beginning (when the patient is ready to be seen by the clinician but none are available) and at the end (due to the use of other services after the clinician is done such as nursing education for preparation for surgery, other ancillary visits such as physiotherapy or genetics, and scheduling of follow-up studies). The decision to use this definition of ACT was due to limited use of the tracking system by clinicians, as opposed to by clinic RNs and registration staff which is more consistent. Additionally, the impact of pre-appointment services on the day of the clinic visit including breast imaging was not evaluated. Lastly, this study only investigated time spent in the breast surgery clinic, and not the total time spent at the institution, whereas patient perception likely includes the latter.

## Conclusions

In conclusion, high-volume clinics and the involvement of trainees prolong the duration of ambulatory clinic encounters. Potential opportunities to mitigate this phenomenon include increasing APP assistance and assigning new patient assessments to trainees versus established patients to attending MDs or APPs. Further investigation of the influence of late patients on encounter times, and the ability to speed up encounters on busy clinic days is warranted. Altering APP work schedules may improve clinician efficiency, but the effects on quality of communication, documentation, and patient satisfaction require further study.

## Data Availability

The datasets used and/or analyzed during the current study are available from the corresponding author on reasonable request.

## Abbreviations

WT: Wait time
ACT: Approximate clinician time
TAL: Total appointment length
APP: Advanced practice provider
IQR: Interquartile range
MRN: Medical record number
MD: Medical doctor
PGY: Post-graduate year
RN: Registered nurse
SPSS: Statistical package for the social sciences
PDSA: Plan-do-study-act
